# Dog Tear Film Proteome In-Depth Analysis

**DOI:** 10.1371/journal.pone.0144242

**Published:** 2015-12-23

**Authors:** Mateusz Winiarczyk, Dagmara Winiarczyk, Tomasz Banach, Lukasz Adaszek, Jacek Madany, Jerzy Mackiewicz, Dorota Pietras-Ozga, Stanislaw Winiarczyk

**Affiliations:** 1 Department of Vitreoretinal Surgery, Medical University of Lublin, 20–079 Lublin, Chmielna 1, Poland; 2 Department and Clinic of Animal Internal Diseases, University of Life Sciences, 20–612 Lublin, Głęboka 30, Poland; 3 Department of Epizootiology and Clinic of Infectious Diseases, University of Life Sciences, 20–612 Lublin, Głęboka 30, Poland; University of Oklahoma Health Sciences Center, UNITED STATES

## Abstract

In this study, mass spectrometry was used to explore the canine tear proteome. Tear samples were obtained from six healthy dogs, and one-dimensional sodium dodecyl sulphate polyacrylamide gel electrophoresis (1D SDS-PAGE) was used as a first step to separate intact proteins into 17 bands. Each fraction was then trypsin digested and analysed by matrix-assisted laser desorption/ionization time-of-flight tandem mass spectrometry (MALDI-TOF-MS/MS) to characterize the protein components in each fraction. In total, 125 tear proteins were identified, with MCA (Major Canine Allergen), Serum albumin, UPF0557 protein C10orf119 homolog, Collagen alpha-2(I) chain, Tyrosine -protein kinase Fer, Keratine type II cytoskeletal, Beta-crystallin B2, Interleukin-6 and Desmin occuring as the most confident ones with the highest scores. The results showed that the proteomic strategy used in this study was successful in the analysis of the dog tear proteome. To the best of our knowledge, this study is the first to report the comprehensive proteome profile of tears from healthy dogs by 1D SDS PAGE and MALDI-TOF. Data are available via ProteomeXchange with identifier PXD003124.

## Introduction

Proteome is a set of proteins expressed in a given time by a given tissue. Its name comes from a blend of proteins and genome. Proteomic analysis has become an important tool in biomedical and veterinary research [1,2,3]. The tear film covering the surface of the eye is a complex body fluid containing thousands of molecules with different structures and functions [3–7]. A molecular analysis of tear film composition is a useful source of information for the diagnosis, prognosis and treatment of diseases of the eye, as well as systemic diseases in humans [8–11]. In addition to its clinical utility, the identification of biomarkers in tear film may be useful in developing new pharmacologically active molecules and diagnostic tests [12–14]. Currently, few publications in the proteomics literature have evaluated the tear film of animals, especially dogs can be of particular interest, as they live in the same conditions and often suffer from diseases of similar aetiopathogenesis [15–17]. Despite well-developed veterinary ophthalmology research concerning dogs, reports on molecular studies of the tear film remains sparse, and in-depth analyses of the protein composition of normal tear film is lacking. Most of the information related to protein profiles was obtained using less-accurate analytical methods [18]. Therefore, a systematic study applying the most advanced proteomic technology should begin with an analysis of the normal tear film protein profile of healthy subjects. This project introduces population studies to determine the correct levels of important tear film proteins in healthy individuals similar to that of haematological standards. The aim of this study was to examine the proteome profile of dog tear samples through one-dimensional sodium dodecyl sulphate polyacrylamide gel electrophoresis (1D SDS-PAGE) in combination with matrix-assisted laser desorption/ionization time-of-flight tandem mass spectrometry (MALDI-TOF-MS/MS).

## Materials and Methods

Tear samples were collected from 6 healthy dogs using a special standard Schirmer’s strip without local anaesthesia. Dogs of various breeds (2 German Shepherds, 1 Doberman, 1 Labrador and 2 mixed breeds) with ages ranging from 2 to 6 years were enrolled during routine admissions to clinics of the Faculty of Veterinary Medicine at the University of Life Sciences in Lublin. Informed consent was obtained from the owners prior to the clinical investigations and sample collection. Every animal used in this study was submitted to a comprehensive ophthalmic examination (anterior segment and fundus evaluation with introcular pressure measurement). Animals included in the study did not exhibit any ocular signs of disease. The exclusion criteria included the presence or history of any systemic or ocular disorder or condition (including ocular surgery, trauma, and disease) that could possibly interfere with the interpretation of the results. The current or recent use of topical ophthalmic or systemic medications that could affect tear status was also grounds for exclusion from this study. The results from blood-cell counts, sera biochemistry and urinalyses oscillated within the normal range. After collection, the Schirmer’s strips were placed in elution buffer consisting of 50 mM phosphate-buffered saline (PBS) with protease inhibitors at 4°C for a maximum of 20 h. The total protein concentration was determined by Bradford’s method at a wavelength of 280 nm (Picodrop, Cambridge, UK). The resulting protein solution was concentrated using SpeedVac at -4°C to a final protein concentration of 60 μg/10 μl.

### Electrophoresis

**Fig 1:**
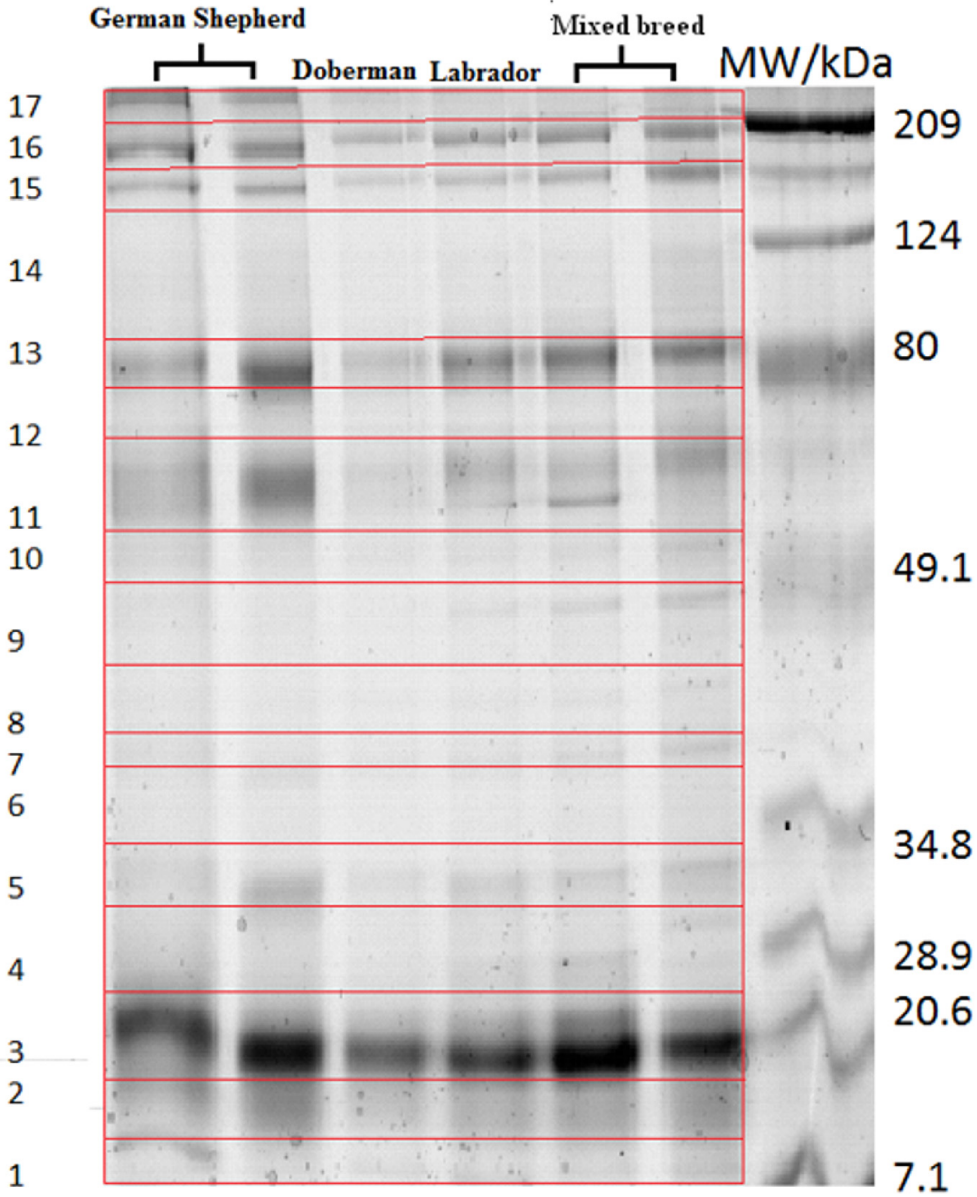
1D SDS–PAGE of Coomassie-stained proteins. Lanes 1–6 are 20 μg of total tear film protein from individual dogs precipitated from tear film collected by a Schirmer strip. Lane 7 is the molecular weight marker.

The protein samples were reduced in dithiothreitol (DTT) (Invitrogen, Carlsbad, CA, USA, cat. no. NP0004), and after mixing with loading buffer (Invitrogen, cat. no. NP0007) and heating to 70°C for 10 minutes, each sample containing 60 μg protein was loaded into a well and subjected to SDS-PAGE analysis using commercial 12% polyacrylamide gel (Invitrogen, NuPAGE^®^ Novex^®^ 12% Bis-Tris). Samples were electrophoresed at 150 V/50 mA/7.5 W until the stain reached 0.5 cm from the edge of the gel. Standard molecular weight markers ranging from 7.1 kDa to 209 kDa were run at the same time. Protein bands were detected by Coomassie Colloidal Blue staining according to the manufacturer’s protocol (Novex, cat. no. LC 6025). In the next step, the lanes were divided into 17 bands, which were excised (Fig 1). Bands of the same molecular mass originating from 6 individual dogs were pooled. The bands that were cut from the 1D gel underwent washing followed by reduction and alkylation using DTT and iodoacetamide. Digestion with trypsin occurred in 50 mM ammonium bicarbonate buffer at 37°C for 12 hours (Promega, Trypsin Gold, Mass Spectrometry Grade, Technical Bulletin). The obtained peptides were sequentially eluted from the gel using a solution of 5%, 10%, 15%, 20%, 25%, 30%, 35%, 40%, 45%, 50% acetonitrile in 5% trifluoroacetic acid (TFA) (v/v). The extracted peptides were purified using μ C18 Zip-TIP pipette tips in accordance with the manufacturer's procedure (Merck Chemicals, Billerica, MA, USA, PR 02358, Technical Note) and applied to the plate MTP AnchorChip 384 (Bruker, Bremen, Germany).

### Mass spectrometry

MALDI was used as a soft ionization method because it only produces a charge and does not cause fragmentation of the analysed compound. The experiment was conducted in an ultrafleXtreme (Bruker) machine with a TOF/TOF detector to guarantee high accuracy and resolution of the measurements. All of the spectra were collected within the 800–3500 Da range in the active reflection mode, and this mass range was used to acquire the MS/MS spectra. HCCA (alpha-cyano-4-hydroxycinnamic acid, portioned; Bruker) was used as the matrix in the dried droplet method (0.5 μl sample + 0.5 μl matrix) following the standard manufacturer’s protocol for peptide analysis. An MTP AnchorChip 384 (Bruker) with hydrophilic spots was used as the holder for sample preparation. Each sample was spotted onto 3 different active spots, and the profiled spectra were calibrated using the peptide mixture Peptide Calibration Standard I (Bruker). The flexControl program 3.3 (version 108) was used for mass spectra collection, flexAnalysis 3.3 (version 80) was used for analysis, and finally, SwissProt database was searched using the software BioTools 3.2 (version 4.48). All spectra were systematically processed as follows: smoothing was performed by the Savitsky-Golay method; baseline subtraction was performed by the Top Hat baseline algorithm; peak geometry was characterized by the Stanford Network Analysis Platform (SNAP) algorithm; and all peaks with a signal ratio above 4 were qualified for further analysis. The parameters for the Mascot database search were as follows: errors in both MS and MS/MS mode at 0.3 Da [19]; global modification of carbamidomethyl (C); possible modification and oxidation (M) [20]; partials at 1; and trypsin enzyme. Spectra with peptide matches above 5 peaks were considered statistically significant, and only 5 proteins were identified with a single peptide match. All of the peptide mass fingerprint spectra were analysed again in MS/MS mode to confirm their exact amino acid sequence.

## Results and Discussion

For over two decades tear film has become an intesivly investigated material due to its assets, like ease to obtain and handle, unlike the other body fluids, ie plasma. The greatest limitation is yet to be the small volume of sample, and low protein concentration. In veterinary field, Hemsley et al were one of the first to investigate tear film for certain proteins, and succeded to find 6 reproducible HPLC protein peaks in dogs, coming to conclusion that they do not correspond in all respects to human tears [18]. With the development of the proteomic approaches like MALDI-TOF, new opportunities arised [21]. De Freitas et al. performed 2-D electrophoresis analysis combined with MALDI-TOF protein identification to find potential cancer biomarkers in dog tear film. They identified some most abundant proteins like MAC, and pointed at albumin and actin elevated levels in dogs with cancer [16]. However there has been no exact identification of each protein present in the electrophoresis, and the samples were collected using microcapillaries, contrary to our Schirmer strips which has been proved to provide more proteins into analysis [22]. Differences with human proteome, similar to those described by de Freitas, like absence of zinc-alpha-2-glycoprotein, were also observed in our study.

MS has become the detection method of choice in proteomics analysis. The majority of studies use a bottom-up approach in which proteins are proteolytically digested into peptides and then subjected to multidimensional protein identification technology. Acquired MS (peptide masses) and MS/MS (sequence information) spectra are used to identify the corresponding proteins via database search algorithms [14]. Based on this proteomics approach, normal human tear fluid was observed to contain almost 500 proteins, although the recent work of Zhou et al. reported that the total number of proteins can reach over 1500 [21]. In our study, we separated intact tear proteins by 1D SDS-PAGE prior to detection using the MALDI-TOF technique. A total protein amount of 60 μg was loaded in each lane of the gel to standardize the sample and ensure that the differences noted in the gel patterns were caused by differences in the presence/absence of proteins rather than other reasons. From the 17 bands, a total of 125 distinct/unique proteins were identified (Tables 1 and 2). Several proteins were observed multiple times in different molecular weight regions of the gel. For example, desmin has a molecular weight of 53 kDa, but it was also observed in bands 15, 16 and 17 between approximately 140 and 210 kDa. Such multiple appearances most likely represent posttranslational modifications or the formation of homopolymers (e.g., dimers, trimers, and multimers of a protein) of lower molecular weight proteins, although they could also represent protein complexes that were not denatured. Higher molecular weight proteins were also observed at lower molecular weight regions in the gel; for example, the collagen alpha-2(I) chain was observed in band 13 at approximately 80 kDa, which might have resulted from protein degradation caused by storage or tear proteases. The human tear protein profile revealed similar variation when analysed by fractionation in 1D electrophoresis or high-performance liquid chromatography (HPLC) in combination with MS [22,23]. Studies of tear film proteins among domestic animals have also shown significant variations [24,25]. Therefore, the accurate and sensitive characterization of tear components in individual species to establish normal tear profiles is crucial for interpreting disease-induced changes, and characterising differences between normal and diseased animals should enhance our understanding of host responses to numerous agents and improve diagnoses, treatments and prognoses. To the best of our knowledge, this study is the first to report the comprehensive proteome profile of tear film from healthy dogs based on 1D SDS-PAGE and MALDI-TOF. In Table 1, we present 125 identified proteins, including the accession number, score, peptide matches, gene name, molecular function and biological process. Certain proteins are described as unclassified because of a lack of information or multiplicity of function. The proteins identified here were classified using the Uniprot.org database according to biological processes and molecular functions. Several abundant tear proteins, such as MCA (Major Canine Allergen), Serum albumin, UPF0557 protein C10orf119 homolog, Collagen alpha-2(I) chain, Tyrosine -protein kinase Fer, Keratine type II cytoskeletal, Beta-crystallin B2, Interleukin-6 and Desmin, as the most confident ones with the highest scores were observed. Some of them hold potential to be used in future as a biomarkers for given diseases, i.e serum albumin in tears is usually weakly expressed, but in human patients with cancer it tends to be highly elevated due to the plasma leakage. One of the most important biological functions of tear proteins is its antimicrobial activity against pathogens because the ocular surface is constantly exposed to the environment [14]. This function is reflected by the substantial representation of immune response proteins, such as cytokines, hydrolases, lysozyme and IgG heavy chains, and a number of these proteins, such as IgG, may be involved in microorganism aggregation rather than death or inhibition. According to Zhou at al., there are top four, well-known human tear film proteins: lysozyme, lactoferrin, secretory IgA and lipocalin [21]. Interestingly, apart from proteins such as lysozyme or serum albumin, many proteins are similar in dogs and humans, including MAC, a main protein found in dog tear film that is most likely analogous to lipocalin, which is found in human tears. However in our study there has been neither presence of sIgA nor lactoferrin in dog tear film. Nevertheless, a number of other proteins are similar or identical in both tear films, like scavenger receptor cysteine-rich type 1 protein M130, which appears to be involved in pattern recognition receptors (PRRs) in humans. All in all, we have revealed that 25 out of 125 proteins identified in our study are common for dogs and humans (Table 3)

**Table 1:** Summary of protein identification.

No.	*Protein*	Accession no.	Score	Matches	Gene name	Molecular function	Biological process
1	*Tuftelin-interacting protein 11*	Q29RR5	35	12	TFIP11	DNA binding	mRNA processing
2	*Selenocysteine insertion sequence-binding protein 2-like*	Q93073	34	4	SECISBP2L	RNA binding	unclassified
3	*Visual system homeobox 2*	P58304	51	6	VSX2	DNA binding	transcription
4	*Tensin-1*	Q9HBL0	61	11	TNS1	RNA binding	unclassified
5	*Zinc finger protein 780A*	O75290	34	4	ZNF780A	DNA binding	transcription
6	*Putative homeodomain transcription factor 1*	Q9UMS5	32	5	PHTF1	DNA binding	transcription
7	*Transcription factor TFIIIB component B'' homologue*	A6H8Y1	40	14	BDP1	DNA binding	transcription
8	*Hepatoma-derived growth factor*	Q8VHK7	37	5	Hdgf	DNA binding	transcription
9	*Metastasis-associated protein MTA3*	Q924K8	32	9	Mta3	DNA binding	cell cycle
10	*Nuclear protein 14*	Q8R3N1	33	13	Nop14	RNA binding	rRNA processing
11	*Zinc finger protein 582*	Q96NG8	34	9	ZNF582	DNA binding	transcription
12	*Zinc finger protein 2*	P08043	61	6	Zfp2	DNA binding	transcription
13	*Eukaryotic translation initiation factor 3 subunit H*	Q91WK2	54	7	Eif3h	RNA binding	protein biosynthesis
14	*78 kDa glucose-regulated protein//Heat shock 70 kDa protein 5*	Q0VCX2	62	10	HSPA5	nucleotide binding	unclassified
15	*Splicing factor*, *proline- and glutamine-rich*	P23246	37	4	SFPQ	nucleotide binding	unclassified
16	*Putative fidgetin-like protein 2*	A6NMB9	41	4	FIGNL2	nucleotide binding	unclassified
17	*UPF0557 protein C10orf119 homolog*	A5PJM5	51	10	MCMBP	chromatin binding	cell cycle
18	*Guanine nucleotide-binding protein G(t) subunit alpha-2*	P38400	30	5	GNAI2	transducer	cell cycle
19	*Protein Spindly*	Q08DR9	54	13	SPDL1	kinetochore binding	cell cycle
20	*Cell division cycle protein 27 homolog*	P30260	41	7	CDC27	phosphatase binding	cell cycle
21	*Kinesin-like protein KIF11*	P52732	55	9	KIF11	motor protein	cell cycle
22	*Parafibromin*	Q6P1J9	44	5	CDC73	RNA polymerase binding	cell cycle
23	*G2/mitotic-specific cyclin-B3*	Q659K0	19	5	CCNB3	cycline	cell cycle
24	*Transcription factor 4*	P15881	28	4	TCF4	activator	transcription
25	*Cysteinyl-tRNA synthetase*, *cytoplasmic//Cysteine—tRNA ligase*, *cytoplasmic*	Q9ER72	38 MS/MS	10	Cars	ligase	protein biosynthesis
26	*Pinin*	P79149	30	4	PNN	activator	transcription
27	*C-C motif chemokine 25*	Q68A93	47	4	CCL25	cytokine	inflammatory response
28	*Interleukin-18*	Q9XSR0	27	3	IL18	cytokine	immune response
29	*Interleukin-12 subunit alpha*	Q28267	41	4	IL12A	cytokine	immune response
30	*Interleukin-1 family member 8//Interleukin-36 beta*	Q9NZH7	46	4	IL36B	cytokine	immune response
31	*Interleukin-6*	P79341	79	7	IL6	cytokine	immune response
32	*Ig heavy chain V-II region COR*	P01815	37	3	N/A	antigen binding	immune response
33	*Scavenger receptor cysteine-rich type 1 protein M130*	Q2VLG6	23	5	CD163	scavenger receptor	inflammatory response
34	*Ig heavy chain V region GOM*	P01784	23 MS/MS	1	N/A	antigen binding	unclassified
35	*Zinc finger BED domain-containing protein 5*	A4Z944	33	6	ZBED5	DNA and metal ion binding	unclassified
36	*Desmoglein-1*	Q9GKQ8	31	5	DSG1	ion binding	cell adhesion
37	*Calcium uptake protein 1*, *mitochondrial*	Q8VCX5	48	7	Micu1	ion binding	calcium transport
38	*Cysteine and glycine-rich protein 2*	Q32LE9	49	5	CSRP2	ion binding	differentiation
39	*Alpha-fetoprotein*	Q8MJU5	25	3	AFP	ion binding	transport
40	*Ankyrin repeat domain-containing protein 5//Ankyrin repeat and EF-hand domain-containing protein 1*	Q9NU02	42	5	ANKEF1	ion binding	unclassified
41	*Zinc finger UFM1-specific peptidase domain protein*	Q3T9Z9	50	7	Zufsp	ion binding	unclassified
42	*N-acetylglucosamine-1-phosphotransferase subunits alpha/beta*	P60529	Protein autopho	4	HBA	oxygen binding	oxygen transport
43	*Haemoglobin subunit gamma*	P02099	60	6	HBG	oxygen binding	transport
44	*Myosin-Ic*	Q63355	56	6	Myo1c	motor activity	transport
45	*Drebrin-like protein*	Q9UJU6	48	6	DBNL	actin binding	transport
46	*Serum albumin*	P49822	60	9	ALB	transport/carrier protein	unclassified
47	*Major allergen Can f 1*	O18873	95, 200 MS/MS	8	N/A	transport protein	unclassified
48	*Gastrin/cholecystokinin type B receptor*	F1Q0L4	40	3	CCKBR	gastrin receptor	unclassified
49	*Growth hormone receptor*	Q9TU69	25	4	GHR	receptor	endocytosis
50	*Transferrin receptor protein*	Q9GLD3	36	8	TFRC	receptor	endocytosis
51	*Gastrin-releasing peptide receptor*	P30550	15 MS/MS	1	GRPR	receptor	cell proliferation
52	*Desmin*	Q5XFN2	87	4	DES	muscle protein	cell structure
53	*Beta-crystallin B2*	P02522	76	6	CRYBB2	eye lens protein	cell structure
54	*Keratin*, *type I microfibrillar 48kDa*, *component 8C-1*	P02534	52	8	N/A	structural	cell structure
55	*Collagen alpha-2(I) chain*	O46392	58	10	COL1A2	matrix protein	cell structure
56	*Adipocytes plasma membrane-associated protein*	Q3T0E5	55	5	APMAP	structural	membrane protein
57	*DnaJ homologue subfamily member 18*	Q5EA26	54	7	DNAJC18	structural	membrane protein
58	*Keratin*, *type II cytoskeletal 8*	Q28810	37	4	KRT8	structural	cell structure
59	*Keratin*, *type I cytoskeletal 9*	P35527	115	17	KRT9	structural	cell structure
60	*Myosin-binding protein C cardiac-type*	O70468	43	13	Mybpc3	structural	cell structure
61	*Collagen alpha-2(IV) chain*	P08122	39	6	Col4a2	matrix protein	angiogenesis
62	*Arylsulfatase K*	Q32KH0	35	5	ARSK	hydrolase	unclassified
63	*Glycogen debranching enzyme*	Q2PQH8	25	9	AGL	hydrolase	glycogen biosynthesis
64	*Cystic fibrosis transmembrane conductance regulator*	Q5U820	29	7	CFTR	hydrolase	ion transport
65	*Lysozyme C*, *spleen isozyme*	P81709	32	3	N/A	hydrolase	antimicrobial
66	*Coagulation factor IX*	P19540	33	5	F9	hydrolase	haemostasis
67	*Proteasome subunit beta type-3*	P33672	54	6	PSMB3	hydrolase	unclassified
68	*Endonuclease 8-like 3*	Q6IE77	57	10	NEIL2	hydrolase	DNA repair
69	*Gamma-glutamyl hydrolase*	A7YWG4	30	6	GGH	hydrolase	unclassified
70	*Multidrug resistance-associated protein 1*	Q6UR05	31	11	ABCC1	hydrolase	transport
71	*Ubiquitin carboxyl-terminal hydrolase 15*	Q9Y4E8	32	6	USP15	hydrolase	transcription
72	*6-phosphofructo-2-kinase/fructose-2*,*6-biphosphatase 2*	O60825	45	5	PFKFB12	hydrolase	unclassified
73	*Probable ATP-dependent RNA helicase DDX58*	Q9GLV6	43	10	DDX58	hydrolase	immune response
74	*ATP-dependent RNA helicase DDX3X*	O00571	40	10	DDX3X	hydrolase	unclassified
75	*Endonuclease 8-like 3*	Q3MHN7	57	10	NEIL3	hydrolase	DNA repair
76	*Werner syndrome ATP-dependent helicase*	Q14191	68	17	WRN	hydrolase	DNA repair
77	*6-Phosphofructokinase*, *muscle type*	P52784	21	2	PFKM	kinase, transferase	glycolysis
78	*Tyrosine-protein kinase Fer*	Q9TTY2	36	6	FER	kinase, transferase	unclassified
79	*Kalirin*	O60229	40	14	KALRN	kinase	unclassified
80	*Cell division protein kinase 3//Cyclin-dependent kinase 3*	Q80YP0	43	4	Cdk3	kinase	cell cycle
81	*Ribosomal protein S6 kinase delta-1*	Q8BLK9	14 MS/MS	1	Rps6kc1	kinase	unclassified
82	*Dual specificity protein kinase CLK3*	P49761	23 MS/MS	5	CLK3	kinase	unclassified
83	*Amine oxidase (flavin-containing)*	Q7YRB7	16	4	MAOB	oxidoreductase	unclassified
84	*Peroxiredoxin-5*, *mitochondrial*	Q9BGI1	57	4	PRDX5	oxidoreductase	unclassified
85	*Cytochrome P450 1A2*	P56592	40	5	CYP1A2	oxidoreductase	unclassified
86	*Lysine-specific demethylase 5C*	Q38JA7	37	12	KDM5C	oxidoreductase	transcription
87	*Lysine-specific demethylase 2B*	Q8NHM5	46, 38 MS/MS	12	KDM2B	oxidoreductase	transcription
88	*Procollagen-lysine*, *2-oxoglutarate 5-dioxygenase 2*	O00469	40	5	PLOD2	oxidoreductase	unclassified
89	*Hydroxysteroid dehydrogenase-like protein 2*	Q2TPA8	42	7	Hsdl2	oxidoreductase	unclassified
90	*Adenine phosphoribosyltransferase*	P08030	37	4	Aprt	transferase	purine salvage
91	*F-box only protein 4*	Q9UKT5	58	7	FBXO4	transferase	cell cycle
92	*Fukutin*	O75072	41	5	FKTN	transferase	unclassified
93	*Chondroitin sulphate synthase 3*	Q70JA7	36	5	CHSY3	transferase	unclassified
94	*Poly[ADP-ribose] polymerase 12*	Q9H0J9	33	7	PARP12	transferase	unclassified
95	*Alkyldihydroxyacetonephosphate synthase*, *peroxisomal*	O00116	31	5	AGPS	transferase	lipid metabolism
96	*Heparan sulphate glucosamine 3-O-sulfotransferase 6*	Q5GFD5	55	6	Hs3st6	transferase	unclassified
97	*Rhophilin-2*	Q8HXG3	22	3	RHPN2	signal transduction	unclassified
98	*F-actin capping protein subunit alpha-2*	Q09YN4	56	5	CAPZA2	actin capping	unclassified
99	*Adenylate cyclase type 5*	P30803	32	7	ADCY5	cyclase	cAMP biosynthesis
100	*Calnexin*	P24643	28	5	CANX	chaperone	protein folding
101	*Rho GTPase-activating protein 7*	B9VTT2	46	10	DLC1	GTPase	signal transduction
102	*Signal recognition particle 68 kDa protein*	Q00004	27	6	SRP68	ribonucleoprotein	unclassified
103	*Endothelin-1*	P13206	34	5	EDN1	vasoactive	unclassified
104	*Signal recognition particle 54kDa protein*	P61010	26	7	SRP54	ribonucleoprotein	unclassified
105	*Oxygen-regulated protein 1*	Q8MJ04	27	13	RP1	microtubule binding	sensory transduction
106	*Arf-GAP with SH3 domain ANK repeat and PH domain-containing protein 1*	O97902	42	7	ASAP1	GTPase activation	cilium biogenesis/degradation
107	*E3 ubiquitin-protein ligase RNF115*	Q9Y4L5	43	4	RNF115	ligase	unclassified
108	*Collectrin*	Q0VCT4	34	4	TMEM27	metalopeptidase	unclassified
109	*EGF-like module-containing mucin like hormone receptor-like 2*	Q2Q421	25	4	2EMR2	unclassified	inflammatory response
110	*Gastrin-releasing peptide*	P47851	55	5	GRP	unclassified	neuropeptide signalling
111	*Protein SDA1 homolog*	Q9NVU7	34	5	SDAD1	unclassified	transport
112	*Sorcin*	P30626	28	3	SRI	unclassified	unclassified
113	*Bcl-2-like protein*	Q9HB09	31	4	BCL2L12	unclassified	apoptosis
114	*Girdin*	Q3V6T2	40, 34 MS/MS	6	CCDC88A	unclassified	DNA replication
115	*Leucine-rich repeat-containing protein 16C*	Q6F5E8	31	6	RLTPR	unclassified	immune response
116	*Coiled-coil domain-containing protein 148*	Q8HZY8	58	9	CCDC148	unclassified	unclassified
117	*COMM domain-containing protein 6*	Q3V4B5	34	4	Commd6	unclassified	unclassified
118	*Breast cancer anti-oestrogen resistance protein 3*	Q9QZK2	40	5	Bcar3	unclassified	unclassified
119	*SH2 domain-containing protein 3C*	Q9QZS8	48	12	Sh2d3c	unclassified	unclassified
120	*Keratin*, *type II cytoskeletal 1*	P04264	112	15	KRT1	unclassified	unclassified
121	*Sestrin-1*	Q4R6P7	55	9	SESN1	unclassified	unclassified
122	*Myelin transcription factor 1-like protein*	P70475	45	6	Myt1l	unclassified	transcription
123	*Coiled-coil domain-containing protein 125*	Q5U465	54	6	Ccdc125	unclassified	unclassified
124	*Phosducin*	O77560	37	5	PDC	unclassified	sensory transduction
125	*Growth arrest-specific protein 6*	Q14393	8 MS/MS	1	GAS6	unclassified	growth regulation

**Table 2:** Proteins found in each band of electrophoretic pattern.

Band	Protein	Mass (kDa)	Score
1	Collectrin	25	34
	Major alergen Can 1	19	90
	Sorcin	22	28
	Bcl-2-like protein	22	31
	E3 ubiquitin-protein ligase RNF115	34	43
	Ig heavy chain V-II region COR	13	37
2	COMM domain-containing protein 6	10	34
	Zinc finger UFM1-specific peptidase domain protein	67	50
	Hepatoma-derived growth factor	27	37
	Major allergen Can f 1	19	95 MS/MS
	Phosducin	29	37
	C-C motif chemokine 25	17	47
	Cysteine and glycine-rich protein 2	22	35
	Adenine phosphoribosyltransferase	20	37
	Signal recognition particle 68 kDa protein	70	26
	Endothelin-1	23	34
	Adipocyte plasma membrane-associated protein	46	55
	DnaJ homolog subfamily C member 18	42	54
3	Ankyrin repeat domain-containing protein 5	87	42
4	Friend of PRMT1 protein	27	10 MS/MS
	Heparan sulphate glucosamine 3-O-sulfotransferase 6	40	55
	Keratin, type II cytoskeletal 8	35	37
	Cell division protein kinase 3	34	43
	Visual system homeobox 2	66	47
	Transcription factor TFIIIB component B'' homolog	300	40
	6-phosphofructo-2-kinase/fructose-2,6-biphosphatse 2	59	42
5	Ankyrin repeat domain-containing protein 5	87	42
	Zinc finger protein 2	54	61
6	UPF0557 protein C10orf119 homolog	74	70
	Calcium uptake protein 1, mitochondrial	55	48
7	Cysteine and glycine-rich protein 2	22	49
8			
	Coagulation factor IX	53	33
	Procollagen-lysine, 2-oxoglutarate 5-dioxygenase 2	86	40
	Ubiquitin carboxyl-terminal hydrolase 15	114	32
9	Dual specificity protein kinase CLK3	74	23 MS/MS
10			
	Parafibromin	61	44
	ATP-dependent RNA helicase DDX3X	74	40
	Haemoglobin subunit gamma	16	60
	Myosin-Ic	120	56
	Zinc finger protein 582	62	34
	Nuclear protein 14	100	33
	Hydroxysteroid dehydrogenase-like protein 2	55	42
	Kinesin-like protein KIF11	120	55
	Metastasis-associated protein MTA3	68	32
	6-Phosphofructokinase, muscle type	86	21
	Tyrosine-protein kinase Fer	95	22
	Keratin, type I microfibrillar 48kDa, component 8C-1	48	52
	Gamma-glutamyl hydrolase	36	30
	Arf-GAP with SH3 domain ANK repeat and PH domain-containing protein 1	126	42
	Ig heavy chain V region GOM	13	23 MS/MS
11	Ribosomal protein S6 kinase delta-1	117	14 MS/MS
	Sestrin-1	57	55
	Myelin transcription factor 1-like protein	135	45
	Serum albumin	70	60
	Beta-crystallin B2	23	76
	Tyrosine-protein kinase Fer	95	23
	Calnexin	68	28
	Rhophilin-2	78	22
	Collagen alpha-2(I) chain	130	58
	Arylsulfatase K	61	28
	Adenylate cyclase type 5	142	24
	Peroxiredoxin-5, mitochondrial	23	57
	Lysozyme C, spleen isozyme	15	32
	Interleukin-18	22	27
	Guanine nucleotide-binding protein G(t) subunit alpha-2	41	30
	Gastrin/cholecystokinin type B receptor	50	40
12	Growth hormone receptor	72	25
	Coiled-coil domain-containing protein 125	57	54
	Werner syndrome ATP-dependent helicase	164	68
	Eukaryotic translation initiation factor 3 subunit H	40	54
	Pinin	88	30
	G2/mitotic-specific cyclin-B3	153	19
	Collagen alpha-2(I) chain	130	44
	Scavenger receptor cysteine-rich type 1 protein M130	127	23
	Gastrin-releasing peptide	15	55
	F-actin capping protein subunit alpha-2	33	56
	Desmoglein-1	115	31
	Cytochrome P450 1A2	58	40
	Endonuclease 8-like 3	62	57
	Interleukin-6	24	79
13	Interleukin-1 family member 8	49	48
	Amine oxidase (flavin-containing) B	59	16
	Transcription factor 4	69	18
	Fukutin	54	41
	Growth arrest-specific protein 6	82	8 MS/MS
	Interleukin-1 family member 8	19	46
	Amine oxidase (flavin-containing) B	59	16
	Selenocysteine insertion sequence-binding protein 2-like	123	34
	Splicing factor, proline- and glutamine-rich	76	37
	Putative fidgetin-like protein 2	67	40
	Protein SDA1 homolog	80	34
	N-acetylglucosamine-1-phosphotransferase subunits alpha/beta	142	30
	Zinc finger protein 780A	77	34
14	Putative homeodomain transcription factor 1	88	32
	Rho GTPase-activating protein 7	126	46
	Signal recognition particle 54kDa protein	56	26
	Alpha-fetoprotein	70	25
	Interleukin-12 subunit alpha	25	41
	Lysine-specific demethylase 5C	177	37
	Pleckstrin	40	32
	Tuftelin-interacting protein 11	96	35
	Transferrin receptor protein 1	87	36
	Protein Spindly	70	54
	SH2 domain-containing protein 3C	95	48
	Keratin, type II cytoskeletal 1	66	112
	Myosin-binding protein C cardiac-type	142	43
	Gastrin-releasing peptide receptor		15 MS/MS
15	Desmin	53	87
	78 kDa glucose-regulated protein	73	62
	Desmin	53	20
	Multidrug resistance-associated protein 1	173	31
	F-box only protein 4	46	58
	Lysine-specific demethylase 2B	155	46, 38 MS/MS
	Kalirin	343	40
	Girdin	217	40, 34 MS/MS
	Tensin-1	187	61
	Chondroitin sulphate synthase 3	101	36
	Leucine-rich repeat-containing protein 16C	156	31
	6-Phosphofructo-2-kinase/fructose-2,6-biphosphatase 2	65	43
	Coiled-coil domain-containing protein 148	73	58
	Probable ATP-dependent RNA helicase DDX58	109	43
	Cysteinyl-tRNA synthetase, cytoplasmic //Cysteine—tRNA ligase, cytoplasmic	95	38 MS/MS
	Collagen alpha-2(IV) chain	108	39
	Breast cancer anti-oestrogen resistance protein 3	93	40
16	Oxygen-regulated protein 1	243	27
	EGF-like module-containing mucin like hormone receptor-like 2	94	25
	Desmin	53	36
	Zinc finger BED domain-containing protein 5	80	28
	Tyrosine-protein kinase Fer	95	36
	Arylsulfatase K	61	35
	Transcription factor 4	69	28
	Adenylate cyclase type 5	142	32
	Glycogen debranching enzyme	177	25
	Cystic fibrosis transmembrane conductance regulator	169	29
17	Proteasome subunit beta type-3	23	54

**Table 3:** Proteins common for human and dog.

No	Protein	Gene
1	Hepatoma-derived growth factor	Hdgf
2	78 kDa glucose-regulated protein//Heat shock 70 kDa protein 5	HSPA5
3	Splicing factor, proline- and glutamine-rich	SFPQ
4	Guanine nucleotide-binding protein G(t) subunit alpha-2	GNAI2
5	Cysteinyl-tRNA synthetase, cytoplasmic//Cysteine—tRNA ligase, cytoplasmic	Cars
6	Interleukin-18	IL18
7	Ig heavy chain V-II region COR	N/A
8	N-acetylglucosamine-1-phosphotransferase subunits alpha/beta	HBA
9	Drebrin-like protein	DBNL
10	Serum albumin	ALB
11	Keratin, type I microfibrillar 48kDa, component 8C-1	N/A
12	Keratin, type II cytoskeletal 8	KRT8
13	Keratin, type I cytoskeletal 9	KRT9
14	Keratin, type II cytoskeletal 1	KRT1
15	Glycogen debranching enzyme	AGL
16	Lysozyme C, spleen isozyme	N/A
17	Proteasome subunit beta type-3	PSMB3
18	6-Phosphofructokinase, muscle type	PKFM
19	Peroxiredoxin-5, mitochondrial	PRDX5
20	Adenine phosphoribosyltransferase	Aprt
21	F-actin capping protein subunit alpha-2	CAPZA2
22	Calnexin	CANX
23	Sorcin	SRI
24	Growth arrest-specific protein 6	GAS6

This shows that animal tear film is similar to human, yet there are some significant differences that have to be taken under consideration during analysis.

These findings may be useful for investigations using dogs as an animal model for certain natural diseases that mimic human disorders.

**Fig 2:**
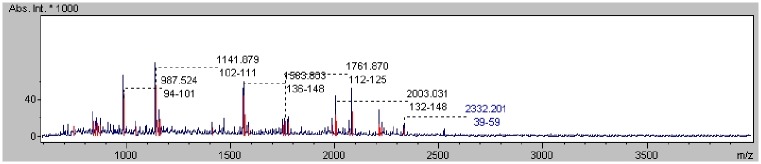
Peptide mass spectra of the major allergen *Canis familiaris* protein.

**Fig 3:**
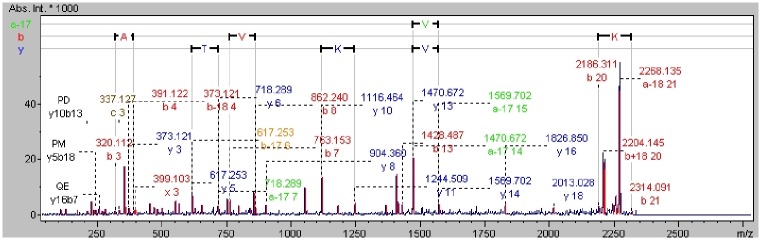
MS/MS peptide mass spectra the 2332.201 m/z peak, which has been identified and confirmed in MS/MS mode as unique for the major allergen *Canis familiaris* protein. The amino acid sequence can be observed on the graph.

The analysis method used to determine the mass spectra of the major allergen *Canis familiaris*, which was also used for the remaining proteins identified in this study, is described below. After acquisition and computation, the protein obtained a score of 78.5 with a statistical significance factor value of 54, and seven peaks with the following masses were assigned to this protein: 987.524 m/z; 1141.879 m/z; 1563.803 m/z; 1586.810 m/z; 1761.870 m/z; 2003.031 m/z; and 2332.201 m/z (Fig 2). The sequence coverage in MS mode was 43.7%. The MS/MS analysis score was equal to 199.87 (987.524 m/z score: 38; 1141.879 m/z score: 22; 1563.803 m/z score: 90; 1586.810 m/z score: 0; 1761.870 m/z score: 43; 2003.031 m/z score: 67; 2332.201 m/z score: 49) with 6 peptide matches and statistical significance factor value 24. (The peak at 1586.810 m/z was rejected as a characteristic of the *Canis familiari*s protein) (Fig 3).

Based on these results, our future work will include two-dimensional (2D) electrophoresis and HPLC in combination with MALDI-TOF-MS and LC-MS/MS with a quadrupole detector for protein identification and sequence characterization. Glycosylation, phosphorylation, and other posttranslational modifications of proteins will be considered during further in-depth analyses.

In summary, we have identified 125 proteins in the tear film of healthy dogs, and to the best of our knowledge, this is the first comprehensive study published thus far. Additional proteomic analysis has been performed by 2D electrophoresis [16]; however, previous studies have not presented a coherent proteome map. Tear film is easily collected non-invasively, and its proteome delivers a rich source of information that may be used for various diagnostics. The mass spectrometry proteomics data have been deposited to the ProteomeXchange Consortium [26] via the PRIDE partner repository [27] with the dataset identifier PXD003124.
